# Molecular dynamics and structure-based virtual screening and identification of natural compounds as Wnt signaling modulators: possible therapeutics for Alzheimer’s disease

**DOI:** 10.1007/s11030-022-10395-8

**Published:** 2022-02-11

**Authors:** Suman Manandhar, Runali Sankhe, Keerthi Priya, Gangadhar Hari, Harish Kumar B., Chetan H. Mehta, Usha Y. Nayak, K. Sreedhara Ranganath Pai

**Affiliations:** 1 Department of Pharmacology, Manipal College of Pharmaceutical Sciences, Manipal Academy of Higher Education, Manipal, 576104 India; 2 Department of Pharmaceutics, Manipal College of Pharmaceutical Sciences, Manipal Academy of Higher Education, Manipal, 576104 India

**Keywords:** Alzheimer’s disease, Wnt signaling, DKK1, LRP6, WIF1, Virtual screening, Molecular dynamics

## Abstract

Wnt signaling pathway is an evolutionarily conserved pathway responsible for neurogenesis, axon outgrowth, neuronal polarity, synapse formation, and maintenance. Downregulation of Wnt signaling has been found in patients with Alzheimer’s disease (AD). Several experimental approaches to activate Wnt signaling pathway have proven to be beneficial in alleviating AD, which is one of the new therapeutic approaches for AD. The current study focuses on the computational structure-based virtual screening followed by the identification of potential phytomolecules targeting different markers of Wnt signaling like WIF1, DKK1, LRP6, GSK-3β, and acetylcholine esterase. Initially, screening of 1924 compounds from the plant-based library of Zinc database was done for the selected five proteins using docking approach followed by MM-GBSA calculations. The top five hit molecules were identified for each protein. Based on docking score, and binding interactions, the top two hit molecules for each protein were selected as promising molecules for the molecular dynamic (MD) simulation study with the five proteins. Therefore, from this in silico based study, we report that Mangiferin could be a potential molecule targeting Wnt signaling pathway modulating the LRP6 activity, Baicalin for AChE activity, Chebulic acid for DKK1, ZINC103539689 for WIF1, and Morin for GSk-3β protein. However, further validation of the activity is warranted based on in vivo and in vitro experiments for better understanding and strong claim. This study provides an in silico approach for the identification of modulators of the Wnt signaling pathway as a new therapeutic approach for AD.

**Graphical Abstract:**

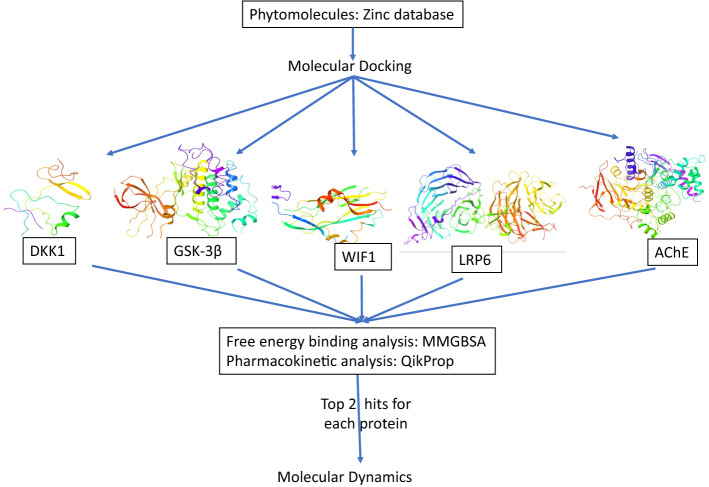

**Supplementary Information:**

The online version contains supplementary material available at 10.1007/s11030-022-10395-8.

## Introduction

Wingless and Integrated (Wnt) [1] comprises a family of secreted glycoproteins that are evolutionarily conserved across various species [2]. The secreted molecules of the Wnt family are involved in the regulation of various essential processes during development like gastrulation, organ development, axis formation, organization of body plan development, and tissue homeostasis [3]. The Wnt signaling pathway plays an important role as a mediator for maintaining intercellular communication that is essential for the development of the nervous system. It is involved in the regulation of neuronal circuits, plasticity, and connectivity by controlling axon remodeling, guidance, morphogenesis of dendrites, and formation of synapses [4].

Continuous generation of new cells occurs throughout adulthood; however, there is a limited capacity for the regeneration of neuronal cells in the adult brain. Recently, the discovery of stem cells in the restricted areas of the brain [5] has brought the possibility of replacing the old dying neurons with stem cells. The activated canonical Wnt/β-catenin signaling pathway is involved in the maintenance of pluripotency of the embryonic stem cells and self-renewal [6]. Members of Wnt signaling pathways play important role in the development and differentiation of stem cells into interneurons [7, 8].

Wnt signaling pathway can be broadly classified into canonical and non-canonical depending on the involvement of β-catenin. Canonical Wnt signaling begins with the binding of Wnt protein to F-class G protein-coupled transmembrane seven helical frizzled receptor and coreceptor lipoprotein receptor-related protein 6 (LRP6). This leads to hyperphosphorylation of disheveled (Dvl) causing the disassembly of the destruction complex comprising of Axin, Adenomatosis polysis coli (APC), and GSK-3β [9–11]. The dissociation of the destruction protein complex inactivates GSK-3β thereby preventing the phosphorylation of β-catenin and its proteasomal degradation. Hence, increased cytoplasmic β-catenin accumulation and its nuclear translocation lead to activation of TCF/LEF target genes [12].

The extracellular protein Dickkopk-1 (DKK1) prevents the interaction of LRP6 with Wnt thereby negatively modulating the canonical Wnt signaling pathway. Similarly, Wnt inhibitory factor (WIF1) is known to inhibit the Wnt signaling as it directly binds to different canonical and non-canonical Wnt ligands Wnt3a, Wnt4, Wnt5a, Wnt7a, Wnt9a, and Wnt11. WIF1 also plays a critical role in the embryonic developmental stage during axis formation. During the early stages of zebrafish brain generation, significant down-regulation of WIF1 and activation of canonical Wnt signaling was observed [13].

Alteration in Wnt pathway leads to age-related diseases like osteoporosis, Parkinsonism, Alzheimer’s disease (AD), and colon cancer [14]. Increased expression of Dkk-1 is seen around the amyloid plaques in the brain of AD patients and the neurons expressing p53 depicting Wnt signaling loss in AD [15]. Downregulated Wnt signaling has been linked to neurodegenerative diseases. Several focused attempts using small molecules and synthetic molecules have been performed to activate the down-regulated signaling pathway for the attenuation of neurodegenerative diseases. Endogenous Wnt-3a ligand-mediated activation of canonical Wnt signaling pathway has proved to provide cell survival signals preventing the neurotoxic effects of amyloid plaques (Aβ) [16, 17]. DKK1 antisense oligonucleotides treatment to the ischemic animals has shown to protect hippocampal neurons against ischemic damage and cultured cortical neurons against NMDA toxicity.

The significance of the computation-based designing of the drug has been proved for several diseases including neurodegenerative disease [18–20]. Natural compounds have immense potential to be converted as novel drugs with specific Wnt signaling targeting [21–24]. In the present study, an in silico-based attempt has been done to screen the phytochemical library from the Zinc database for the evaluation of the binding affinity with different proteins (DKK1, LRP6, WIF1, GSK3β) of Wnt signaling pathway and acetylcholinesterase (AChE).

## Materials and methods

All computational-based studies were performed on the Maestro platform, involving LigPrep, the protein preparation wizard, GLIDE (Grid-based Ligand Docking with Energetics), and Desmond tools by Schrodinger Inc.

### Protein preparation

In structure-based molecular modeling, the use of accurate protein structure is important. Downregulated Wnt signaling has been linked to AD. In the present study, proteins linked with Wnt signaling pathway have been selected to explore the binding affinity, interactions of the selected phytomolecules and virtually evaluate their potential to upregulate Wnt signaling. RCSB PDB id: 2YGO [25], 3S2K [26], 1Q5K [27], and 4M0F [28] were retrieved from protein data bank and prepared using ‘protein preparation wizard’ of Maestro, Schrodinger suite.

WIF1 is known to inhibit the Wnt signaling as it directly binds to different canonical and non-canonical Wnt ligands. The PDB id 2YGO which represents human WIF domain-EGF-like domain 1 of WIF1 in complex with 1,2-dipalmitoylphosphatidylcholine was selected for identifying molecules with good interaction and possible WIF1 inhibiting potential. Increased expression of Dkk-1 is seen around the amyloid plaques in the brain of AD patients and the neurons expressing p53 resulting in the Wnt signaling loss in AD [15]. Inhibition of DKK1 protein or its binding with LRP6 could be a possible therapeutic alternative for AD. Currently, we have chosen PDB id: 3S2K which represents the structural basis of inhibition of Wnt signaling by Dickkopf binding to LRP5/6. It has been proved that Aβ exposure induces overexpression of GSK-3β that mediates hyperphosphorylation of Tau protein and eventually the development of AD [29, 30]. Inhibition of GSK-3β has immense potential as an alternative therapy for AD. PDB id: 1Q5K was selected that represents the crystal structure of GSK-3β in complex with inhibitor n-(4-methoxybenzyl)-n'-(5-nitro-1,3-thiazol-2-yl)urea. PDB id 4M0F represents the crystal structure of human acetylcholinesterase (AChE) in complex with territrem B.

The X-ray crystallographic structures of the above-mentioned proteins (PDB id: 2YGO, 3S2K, 1Q5K, 4M0F) were imported, pre-processed, and minimized using the protein preparation wizard. In these steps, missing side chains and amino acids were filled, heavy atoms and water molecules were removed, and restrained minimization was done to generate the lowest energy protein structure at neutral pH. All the important amino acids are retained in protein structure during protein preparation. For protein GSK-3β, AChE receptor grid was generated based on the co-crystallized ligand using the ‘Receptor grid generation panel’ from GLIDE [31] module, whereas the grid for other proteins was generated based on active druggable sites obtained from the site map [32].

### Ligand preparation

Phytomolecules (1924) from the Biogenic subset of the ZINC database [33] were prepared using the 'LigPrep' module of Maestro, Schrodinger suite. This module is used to generate (i) the lowest energy 3D structures with correct chirality, (ii) tautomers, (iii) ring conformation, (iv) stereochemistry, and (v) ionization states using Epik. The ligand preparation process was performed at neutral pH under the OPLS3e force field.

### Structure-based molecular docking

After ligand preparation, molecular docking was performed using high throughput virtual screening (HTVS), followed by Standard precision (SP) and extra precision (XP) mode, using the GLIDE module of Maestro, Schrodinger suite [34]. Docking of the library of phytomolecules was done for selected five proteins based on the receptor grid which were generated either using inbound ligands for GSK3β and AChE proteins or using the sites generated using sitemap tool. Docking in HTVS mode facilitated in a quick screening of the hit molecules for different proteins (1467 molecules for GSK-3β; 1523 molecules for AChE; 1605 molecules for DKK1; 978 molecules for LRP6; and 1391 molecules for WIF1 protein). Further, based on the dock score and binding interactions, 500 molecules from these shortlisted hit molecules were selected and docked in SP mode of docking, and eventually, the top 200 molecules for each protein were docked using XP mode of docking.

### Binding energy calculation

The relative binding energy of 20 XP docked protein–ligand complexes was calculated with each selected protein, using Prime-Molecular Mechanics-Generalized Born Surface Area (MM-GBSA) [35–37]. Prime MM-GBSA uses the VSGB solvation model [38] which is dependent on the variable-dielectric generalized Born model and solvent as water under the OPLS3e force field. Binding energy also helps in predicting how strongly the ligand binds to the selected protein.

The binding free energy of all the selected proteins–ligand complex is calculated using the formula:

∆G bind = G complex–(G protein + G ligand).

where G = MME (molecular mechanics energy) + GSGB (SGB salvation model for polar solvation) + GNP (nonpolar solvation).

### ADME analysis

The ADME analysis was calculated for the top 20 hits for each protein, using the ‘QikProp’ module of Maestro, Schrodinger suite [39]. The ‘QikProp’ tool helps in predicting the drug-like properties of selected ligands. In this context, the ADME properties such as molecular weight, QPlogPo/w (octanol/water partition coefficient), QPlogS (solubility), QPlog HERG (ability to block K + channel), QPPCaco (Caco2 cell permeability), QPlogBB (Blood/brain partition coefficient), human oral absorption (%oral absorption), and rule of five were calculated.

### Molecular dynamic (MD) simulation

The ‘Desmond’ module of Maestro, Schrodinger suite, was used to carry out MD simulation of top two hits with all five of the selected proteins. In the Desmond module, the orthorhombic simulation box with specific dimensions using an explicit solvent system simulated under different conditions was used. In the current study, two hits for each protein were selected for MD simulation, based on the results obtained from XP docking, binding interactions, and ADME analysis. MD simulation was done in three steps, namely system builder, minimization, and MD simulation. During the first step, the docked protein–ligand complex was subjected to a system builder using a predefined SPC solvent system. The SPC solvent system is mainly used to create orthorhombic boundary conditions. Also, by the addition of sodium ions, the negative charge was neutralized in the model. In the next step, the generated model was subjected to minimization, which is further balanced at 1 bar pressure using NPT (normal pressure–temperature) ensemble and 300 K pressure. The MD simulation was performed for 50 ns, in which for 50-ps frame was captured and saved into trajectory. Overall, 1000 frames were captured during MD simulation.

### Analysis after MD simulation

After MD simulation, calculation of RMSD of protein and ligand as well as analysis of the interactions shown by ligand during entire simulation duration was done. RMSD measures the average change in displacement of selected atoms for a particular frame with respect to a reference frame.

The RMSD for frame x is:

$$ {\text{RMSD}}_{X} \; = \;\sqrt {\frac{1}{N}\sum\limits_{i = 1}^{N} {\left( {r_{j}^{\prime } \left( {t_{x} } \right) - r_{j} \left( {t_{ref} } \right)} \right)^{2} } } $$

where *N* is the number of atoms in the atom selection. *t*_ref_ is the reference time, and *r*׳ is the position of the selected atoms in frame *x*

## Results and discussion

### SiteMap prediction for the drug-binding pocket

For three of the proteins, LRP6 (PDB id: 3S2K chain A), DKK1 (PDB id: 3S2K chain X), and WIF1 (PDB id: 2YGO), the co-crystalized ligand structures were not present. Hence, the SiteMap prediction tool was used to predict the druggable binding site. SiteMap prediction led to the identification of a druggable site of volume 573.496 Ǻ^3^ with the druggability score (Dscore) of 1.291 and site score of 1.204 for protein WIF1. The identified pocket for WIF1 protein comprised of the total surface area of 3821.811 Ǻ^2^ which included hydrophobic region of 526.63 Ǻ^2^, hydrophilic area of 1969.72 Ǻ^2^, and hydrogen bond acceptor region of 600.446 Ǻ^2^. Similarly, for DKK1 protein three druggable sites were identified with the best site as site1 with 307.671 Ǻ^3^ volume, site score of 0.878, and Dscore of 0.998. The identified site 1 pocket for DKK1 protein comprised of the total surface area of 5811.98 Ǻ^2^ which included hydrophobic region of 46.31 Ǻ^2^, hydrophilic area of 3172.79 Ǻ^2^, hydrogen bond acceptor region of 1624.40 Ǻ^2^, and hydrogen bond donor region of 1531.69 Ǻ^2^. Sitemap generated five druggable pockets for protein LRP6 with the best site as site3 of volume 308.7 Ǻ^3^ with Dscore of 1.042 and sites score of 1.147 containing the residues involved in the binding to DKK1. The identified site 3 pockets for LRP6 protein comprised of the total surface area of 871.75 Ǻ^2^ which included hydrophobic region of 83.06 Ǻ^2^, hydrophilic area of 713.35 Ǻ^2^, hydrogen bond acceptor region of 252.79 Ǻ^2^, and hydrogen bond donor region of 468.27 Ǻ^2^. The list of the generated pockets for different proteins with the Dscore, Site scores, and the residues involved in the pocket is represented in Table 1. The grid for docking was generated using the coordinates of the best-identified sites for these three proteins.

**Table 1:** List of identified druggable pockets in the proteins WIF1, DKK1, and LRP6 with the Dscore, Site score, the volume of pocket, and available residues in the pocket

S. no.	Sites	Site Score	D Score	Volume (Å^3^)	Residues
*WIF1*					
1	Site 1	1.204	1.291	573.496	**Chain A**: GLU36, TYR37, LEU38, ILE40, LEU48, ILE49, ILE55, LE57, VAL58, MET63, PHE66, THR67, ASP69, PHE70, ARG71, ALA73, GLN74, GLN75, ARG76, MET77, PRO78, ALA79, ILE80, MET87, PHE89, TRP91, GLN92, TYR101, PHE103, VAL127, AL134, VAL136, PHE138, PHE150, VAL152, VAL154, VAL156, LEU165, THR167, PRO168, ILE172, PHE173, PHE174
*DKK1*					
1	Site 1	0.878	0.998	307.671	**Chain C:** LYS182, GLN184, GLU185, GLY186, LEU190, ARG191, SER192, SER193, CYS195, ALA196, SER197, GLY198, LEU199, CYS201, ALA202, ARG203, HIS204, LYS208, ILE209, CYS210, LYS211, PRO212, VAL213, LEU214, LYS215, GLU216, GLN218, VAL219, CYS220, THR221, LYS222, HIS223, ARG224, ARG225, LYS226, SER228, HIS229, LYS231, GLU232, PHE234GLN235, ARG236, CYS237, TYR238, CYS239, GLU240, GLU241, GLY242, LEU243, SER244, CYS245, ARG246, ILE247, ARG259, LEU260, HIS261, ARG265, HIS266
2	Site 2	0.603	0.668	21.266	**Chain C:** VAL188, CYS189, LEU190, ARG203, PHE205, TRP206, SER207, LYS208, GLY230, ILE233
3	Site 3	0.48	0.496	6.86	**Chain C:** GLU216, GLY217, ARG246, ILE247, GLN248, LYS249, GLN264, ARG265
*LRP6*					
1	Site 1	1.063	1.008	932.274	**Chain A:** THR934, LYS941, ASN966, VAL967, ARG968, ALA969, ILE970, ASP971, TYR972, PRO974, TYR1017, ASP1018, LEU1019, SER1020, ILE1021, ASP1022, ILE1023, TYR1024, ALA1061, ILE1062, VAL1063, VAL1064, ASN1065, PRO1066, GLU1067, ILE1105, ALA1106, LEU1107, ALA1108, LEU1109, ASP1110, SER1111, ARG1112, LEU1113, GLN1146, VAL1148, GLY1149, LEU1150, THR1151, VAL1152, PHE1153, GLU1154, LYS1162, GLN1187, LEU1188, SER1189, ASP1190, ILE1191, HIS1192, ALA1193,VAL1194, LYS1195, GLU1196, LEU1197, ASN1198, GLU1201, TYR1202
2	Site 2	0.994	0.984	695.947	**Chain A:** HIS698, VAL699, VAL700, GLU701, PHE702, GLY703, TRP721, THR726, ARG728, GLU730, GLY736, GLN737, HIS738, ARG739, VAL741, TRP744, HIS902, PRO917, ALA918, TYR920, ALA931, PRO932, THR933, PHE935, GLU938, GLN940, ASN945, ARG946, MET947, VAL948, ILE949, ASP950, GLN953, SER954, PRO955, ASP956, ILE957, ILE958, LEU959, PRO960, TYR972, PRO974, LYS977, GLU993, GLU994, ASP995, GLN1182, ILE1185, ALA1186, VAL1194, LYS1195, GLU1196, LEU1197, LEU1199
3	Site 3	1.147	1.042	308.7	**Chain A:** SER665, ALA666, LEU667, ASP668, PHE669, GLU708, GLY709, MET710, ALA711, VAL712, TRP714, ARG751, ALA752, LEU753, ALA754, LEU755, TRP767, ASN794, GLY795, LEU796, THR797, ILE798, TYR800, LEU810, PHE836, GLY837, LEU838, THR839, MET877, ASP878, ILE879, LEU880, VAL881, ARG886, GLN887
4	Site 4	0.974	0.976	373.184	**Chain A:** GLN740, VAL741, LEU742, VAL743, TRP744, LYS745, ASP779, GLY780, SER781, GLU782, ARG783, LEU905, VAL913, CYS914, GLY915, CYS916, PRO917, ALS918, HIS919, TYR920, SER921, LEU922, PRO932, THR933, THR934, PHE935, VAL948, ILE949, ASP950, GLN952, SER954, PRO955, PHE1153
5	Site 5	0.885	0.868	206.486	**Chain A:** TRP714, ASP756, ALA758, GLU759, PHE761, TYR763, ARG775, ASP799, TYR800, ALA801, ARG803, MET820, GLN840, TYR841, GLN842, SER885, ARG886, GLN887, SER888, GLY889, VAL909

Red color indicates hydrogen bond acceptor region, blue color indicated hydrogen bond donor region, and yellow color indicates a hydrophobic region of the identified sites

### Docking protocol validation

**Fig. 1:**
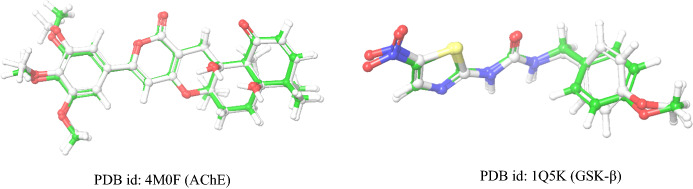
Superimposition of redocked and the co-crystalized ligand in the protein 4M0F and 1Q5K for the validation of docking protocol

The validation of the grid generated was done by redocking the co-crystalized ligand using the generated grid followed by superimposition and comparison of the RMSD. The redocking was performed followed by alignment with the aligned poses as shown in Fig. 1. Docking with the inbound inhibitor of PDB id: 4MOF showed the docking score of − 13.022 kcal/mol with H-bond interaction with residue TYR 124, PHE295, and π–π stacking interaction with TRP286. Docking of the co-crystalized ligand of PDB id: 1Q5K showed a dock score of − 7.556 kcal/mol with the formation of H-bond interaction with LYS85 and VAL135 residues. The superimposition of the docked ligand with the co-crystalized ligand showed RMSD of 0.55 in 4M0F and 0.706 in 1Q5K indicating that grid generation occurred in the desired pocket as that of the inhibitor binding site.

### Virtual screening of the selected phytochemicals

In the current study, we have listed, analyzed, and explained the dock score, and binding interactions of the top five ligands for each protein after docking in XP mode of docking.

### Molecular interaction of top five ligands with acetylcholinesterase

AChE enzyme is involved in the regulation of the level of the neurotransmitter, acetylcholine mediated through the hydrolysis of acetylcholine into choline and acetate. In AD, the level of acetylcholine neurotransmitters is reduced. FDA-approved AChE inhibitors act by increasing the level of acetylcholine in the brain. The active site for human AChE has shown a deep gorge of 20Ǻ depth with the catalytic triad formed by residues HIS447, GLU334, and SER203 [28]. Donepezil–AChE complex shows the formation of interaction between benzyl ring of donepezil with TRP86; indanone ring interacting with TRP286 residue [40]. In the current study, for evaluation of the binding affinity of the phytochemicals to AChE protein, PDB id: 4M0F grid was generated by considering the coordinates of the co-crystalized ligand territrem B. The binding site of territrem-B comprises both catalytic and peripheral sites involving π–π stacking interaction between side chains of TRP 286 and TYR341; hydrogen bond interaction with residues SER293, TYR72, and TYR124 [28].

Currently, in this study, we found the top docking score of − 14.205 kcal/mol shown by Mangiferin. Mangiferin, a xanthone member consisting of 1,3,6,7-tetrahydroxyxanthen-9-one and a beta-D-glucosyl residue at the 6-position, is present at a significant level in the mango fruit, peel, leaves, and kernel. Glucosyl residue of Mangiferin showed the hydrogen bond interactions with amino acid residues SER203, and HIS447 involved in the triad of the active site and xanthone ring formed π–π interaction with TRP286 residue as shown by the standard known AChE inhibitors. Similarly, Baicalin showed the second highest dock score of − 13.224 kcal/mol. Baicalin (5,6,7-trihydroxyflavone) belongs to the flavone class of glycoside chiefly found in the herb *Scutellaria baicalensis.* Hydroxyl group of 7-O-glucuronide of baicalin forms hydrogen bond interactions with ASP74, Similarly, hydroxyl and oxy group of benzopyran ring of baicalin forms H-bond interaction with TYR 341and PHE295 residues, respectively; benzene ring of benzopyran ring is involved in the formation of π–π interaction with TRP286. The docking scores of top molecules were found to be in the range of − 14.205 to − 10.987 kcal/mol. The list of the top two ligands along with their dock score, binding energy, and interactions is shown in Table 2.

**Table 2:** Dock score, binding energy, and interacting residues with 2D representation for Acetylcholinesterase enzyme (PDB id: 4M0F), LRP6, DKK1 protein (PDB id: 3S2K), GSK-3 β enzyme (PDB id: 1Q5K), WIF1 protein (PDB id: 2YGO) of top two molecules

Compound	XP Dock score (Kcal/mol)	MMGBSA dG Bind (Kcal/mol)	Interactions	2D Interaction diagram
*Extra precision Molecular docking for AChE (PDB id: 4M0F)*				
ZINC33832403 (Mangiferin)	− 14.205	− 52.65	**H-Bond:** TYR72, SER203, HIS447 **π**–**π interaction:** TRP286 **Hydrophobic:** TYR72, LEU76, TRP86, TYR124, ALA204, TYR286, PHE297, TYR337, PHE338, TYR341 **Polar:** THR75, HIS447, SER203 **Charged Negative**: ASP74, GLH202	
ZINC3943903 (Baicalin)	− 13.224	− 43.28	**H-Bond:** ASP74, PHE295, TYR341 **π**–**π interaction:** TRP286 **Hydrophobic:** TYR72, LEU76, TRP86, TYR124, TRP286, VAL294, PHE295, PHE297, TYR337, PHE338, TYR341 **Polar:** THR75, SER293, HIS447 **Charged Negative:** ASP74	
*Extra precision Molecular docking for LRP6 (PDB id: Chain A _3S2K)*				
ZINC33832403 (Mangiferin)	− 10.960	− 44.19	**H-Bond:** ASP668, GLU708, MET710, LEU753, LEU755, TYR800, THR797 **Hydrophobic:** PHE669, ALA666, MET710, ALA711, VAL712, TRP714, ALA752, LEU753, ALA754, LEU755, LEU796, ILE798, TYR800, LEU838, MET877, ILE879, LEU880 **Polar:** ASN794, THR797, THR839, GLN840, GLN887 **Charged Negative:** ASP668, GLU708, ASP878 **Charged Positive:** ARG886	
ZINC12504453 (Calystegine)	− 10.487	− 32.32	**H-Bond:** LEU667, MET710, LEU753, LEU838, ASP878 **Salt Bridge:** ASP878 **Hydrophobic:** LEU667, ALA666, MET710, ALA711, ALA752, LEU753, ALA754, LEU796, LEU838, ILE879, LEU880 **Polar:** ASN794, THR797, THR839 **Charged Negative:** ASP668, ASP878	
*Extra precision Molecular docking for DKK1(PDB id: Chain X_3S2K)*				
ZINC33832403 (Mangiferin)	− 11.155	− 48.53	**H-Bond:** SER192, CYS201, LYS208, HIE229, THR221 **Hydrophobic:** LEU190, CYS200, CYS201, ALA202, CYS220, CYS237, TYR238, CYS245 **Polar:** SER192, THR221, HIS223, HIE229, GLN235 **Charged Positive:** ARG191, ARG203, LYS208, LYS222, ARG236	
ZINC000013385490 (Chebulic acid)	− 9.972	− 34.28	**H-Bond:** THR221, HIS223, HIE229, GLN235, ARG236, CYS239 **Salt bridge:** LYS222 **Hydrophobic:** LEU214, CYS220, CYS237, TYR238, CYS239, CYS245 **Polar:** THR221, HIS223, HIE229, GLN235 **Charged Negative:** LYS222, ARG236	
*Extra precision Molecular docking for GSK-3β (PDB id: 1Q5K)*				
ZINC33832403 (Mangiferin)	− 10.344	− 34.91	**H-Bond:** ASP133, LYS183, ASN186, ASP200 **Hydrophobic:** ILE62, PHE67, VAL70, ALA83, VAL110, LEU132, TYR134, VAL135, LEU188, CYS199 **Polar:** SER66, GLN185, ASN186 **Charged Negative:** ASP133, ASP181, ASP200 **Charged Positive:** LYS85, LYS183	
ZINC3881558 (Morin)	− 9.427	− 41.27	**H-Bond:** VAL135 **Hydrophobic:** ILE62**,** VAL70, ALA83, VAL110, LEU132, TYR134, VAL135, PRO136, LEU188, CYS199, **Polar:** THR138 **Charged Negative:** ASP133, GLU137, ASP200 **Charged Positive:** LYS85, ARG141	
*Extra precision Molecular docking for WIF1 (PDB id: 2YGO)*				
ZINC33832403 (Mangiferin)	− 13.546	− 53.75	**H-Bond:** PRO78, THR167 **π**–**π interaction:** PHE89, PHE173 **Hydrophobic:** LEU38, ILE40, LEU48, ILE49, ILE57, MET63, PHE70, MET77, PRO78, ILE80, MET87, PHE89, PHE138, VAL136, PHE150, VAL152, VAL154, PRO168, PHE173, **Polar:** THR167	
ZINC103539689	− 12.667	− 60.85	**π**–**π interaction:** PHE173 **Hydrophobic:** LEU38, ILE40, LEU48, ILE49, ILE55, ILE57, MET63, PHE66, PHE70, MET77, PRO78, ALA79, ILE80, MET87, PHE89, PHE103, VAL136, PHE138, PHE150, VAL152, VAL154, PRO168, PHE173, PHE174 **Polar:** THR167 **Charged Positive:** ARG76	

### Molecular interaction of top five ligands with LRP6

LRP6 acts as a coreceptor for Wnt ligands that along with the Frizzled receptor activates the Canonical Wnt/β-catenin signaling pathway. Sequence analysis has revealed that LRP6 proteins are composed of four repeats of extracellular YWTD domain (P1-P4) paired by Epidermal growth factor (EGF)(E1-E4) repeats ranging from amino acid 21 to 1246 followed by three LDLR type domains [41]. The four repeats formed by YWTD and EGF (1–4) represent the functional recognition ectodomain involved in the binding with Wnt and antagonists. Based on the studies, the tandem repeats can be broadly divided into LRP6(1–2) And LRP6(3–4) units. LRP6(1–2) has been proved to be a binding site for Wnt9b, Wnt1 while site LRP6(3–4) favors WNt3a [17]. DKK1-mediated inhibition of LRP6 is based on binding of DKK1 to the third and fourth β-propeller domain of LRP6 (P3E3P4E4) which extends from 630 to 1244. The crystal structure of the DKK1-LRP6 complex confirms the top surface of LRP6 as the binding site of DKK1. Mutagenic and stoichiometric experiments have reported that DKK1C forms 1:1 complex with LRP6 and validated LRP6 P3 as the binding site for DKK1c mediated Wnt signaling inhibition [42]. A study done by Chen et al*.,* to understand the effect of the single point mutation in LRP6 and its binding affinity with DKK1 suggests the role of specific residues in the binding with DKK1 [43]. Single-point mutants TYR706A, GLU708A, and TRP767A of LRP6 have resulted in the reduced binding to DKK1 suggesting the direct role of these residues in the participation with DKK1 interaction. Residues GLU663, ILE681, TYR708, ASP748, SER749, ARG751, TRP767, GLY769, ARG792, ASN 794, LEU810, ASP811, ASP830, TRP850, and MET877 of LRP6 are involved in the interaction with DKK1 protein.

Hydroxyl groups in glucosyl moiety of mangiferin showed the hydrogen bond interactions with ASP668, LEU755, TYR800, and hydroxyl and oxy groups attached to xanthone ring of mangiferin formed H-bond interactions with GLU708, MET710, LEU753, and THR797 indicating that mangiferin binds in the same pocket as that of DKK1. Similarly, compound calystegine showed a docking score of − 10.487 kcal/mol with its hydroxyl groups forming hydrogen bonds with residues LEU667, MET710, LEU753, LEU838, ASP878, and the amine group of azabicycloctane group of calystegine formed a salt bridge with ASP878. The top five compounds showed dock scores in the range of − 10.694 to − 8.913 kcal/mol and free binding energy from − 44.19 to − 22.38 kcal/mol. Top two compounds with dock score, interacting residues, and binding energy are listed in Table 2.

### Molecular interaction of top five ligands with Dickkopf-1 (DKK1) protein

DKK1 is an extracellular secretory protein with high affinity for LRP6, and Kremen, promoting endocytosis of LRP6 followed by its degradation making LRP6 unavailable for Wnt signaling [44]. Proteins of Dkk family include cysteine-rich domains Dkk_N and Dkk_C that are linked with a linker of around 50 residues.DKK1, Wnt antagonist binds to both of the sites DKK1_N to LRP6(1–2) and DKK1_C to LRP6(3–4) [26]. An experiment-based study by Gregory et al. has reported the second Cysteine-rich domain DKK_C comprising of residues (CYS217-ARG237; CYS233-CYS253) to be involved in the inhibition of the Wnt signaling pathway [45]. Based on the experiment done by Rismani et al., in-hotspot region selection by alanine scanning the binding site residues GLN184, HIS204, TRP206, ILE209, LYS211, VAL219, CYS220, THR221, LYS222, ARG224, ARG236, ARG259, and LEU260 in DKK1 was identified to form interaction with LRP6 protein [18]

Currently, in our study, the top site generated by the sitemap tool with the best site score and Dscore including the important residues as suggested by the experimental and computational-based method was selected for the screening of the phytomolecules. Mangiferin has shown a top dock score of − 11.15 kcal/mol among all the molecules with the hydroxyl groups in its glucosyl moiety forming hydrogen bonding with residues SER192, CYS201, and LYS208. Hydroxyl groups and oxy groups of xanthone ring formed H-bond interactions with THR221 and HIE229 residues. Chebulic acid, a phenolic compound isolated from the ripened fruits of *Terminalia chebula*, showed the second highest dock score of − 9.972 kcal/mol. Hydroxyl groups attached to isochroman ring of chebulic acid from H-bond interactions with the residues HIE229, GLN235, ARG236, THR221, carboxyl group formed H-bond interactions with HIS223, CYS239, and salt bridge formation with LYS222. Interaction with residues THR221 and ARG236 was found common in most of the top hit molecules. The detailed information of 2D ligand interaction, docking score, and binding energy of two top hit molecules is listed in Table 2.

### Molecular interaction of top ligands with GSK-3β protein

The involvement of the GSK-3β has been well established in neuropathological disorders like amyloid deposition, gliosis, and tau hyperphosphorylation. Tideglusib, ATP non-competitive GSK3β inhibitor, has completed phase II of the clinical trial and has been recognized as an orphan drug for the treatment of rare tauopathy by the Food and Drug Administration (FDA). GSK-3β contains two major domains: N-terminal β-strand domain extending from the amino acid residues 25–138 and an α-helical C-terminal domain with residues 139–343. The interface of these two domains contains an ATP-binding site that is connected by a glycine-rich loop and hinge region. The ATP binding pocket involves residues LYS85, GLU97, ASP113, TYR134, VAL135, THR138, ASN186, LEU188, CYS199, ASP200 [46]. The co-crystallized structure of GSK-3β with its inhibitor AR-A014418 reveals that the inhibitor occupies the hinge region along with the ATP pocket through the formation of three hydrogen bonds with residue VAL135. GSK-3β catalytic activity is regulated by phosphorylation at SER9 and TYR216 residues. Phosphorylation of the SER9 site inactivates GSK-3β, whereas phosphorylation at TYR216 within the activation loop increases its catalytic activity [47].

Currently, screening of phytochemicals with virtual docking study for GSK-3β enzyme resulted in the identification of mangiferin as the hit molecule. Hydroxyl groups in the glucosyl moiety of mangiferin showed hydrogen bond interaction with amino acid residues LYS183, ASP200, and salt bridge interaction with ASN186 residue with an XP score of − 10.344 kcal/mol. Hydroxyl group of xanthone ring of mangiferin formed H-bond interaction with ASP133 residue. Morin, a pentahydroxy flavone has been proved to possess antioxidant, antihypertensive, neuroprotective activity. The hydroxyl group of morin showed two hydrogen bond interactions with the amino acid residue VAL135. From the binding interactions of the hit molecules with the protein, we observed the residues VAL135 and ASP200 to be common and important. The detailed information of 2D ligand interaction, docking score, and binding energy of the top two hit molecules is listed in Table 2, and the top 5 hit molecules have been listed in the supplementary file.

### Molecular interaction of hit molecules with WIF1 protein

WIF1 binds to Wnt proteins thereby, preventing the binding of Wnt to Frizzled receptor and inhibiting the Wnt signaling pathway. WIF-1 consists of N-terminal region, WIF domain (38–177 amino acid residue), five EGF-like domains each with 31–33 residues extending from 178 to 338 residues, and hydrophilic C terminal. Based on site-directed mutagenesis, biophysical and cell-based in vitro assays Malinasuskas et al*.* have revealed the involvement of both WIF domain and EGF domain in the binding of Wnt. Their mutagenesis study to reveal the involvement of areas in WIF domain for recognition of Wnt3a found that the mutation in residue MET77 was observed to show 10 times decrease in the binding affinity to Wnt3a [25].

In the present study, docking of natural phytomolecules led to the identification of mangiferin as the hit molecule showing strong binding with WIF1 protein with dock score of − 13.546 kcal/mol. Glucosyl residue of mangiferin hydrogen bonding interaction with amino acid THR167 and the hydroxyl group of xanthone ring of mangiferin formed H-bond with PRO78 residue, π–π interaction with PHE89 and PHE167 residues of WIF1 domain. MET77 residue formed the boundary and nonbonding interaction for the top hit molecules. ZINC103539689 molecule showed dock score of − 12.667 kcal/mol and formed π–π interaction with residue PHE173. Other hit phytomolecules showing stable binding energy of − 34.70 to − 71.13 kcal/mol and docking scores ranging from − 12.680 to − 9.756 kcal/mol. Top two hit molecules with the interacting residues are listed in Table 2.

### MMGBSA analysis for binding free energy calculation

Binding free energy was calculated and tabulated for all the top five compounds from docking simulations. The top molecules for AChE showed binding energy in the range of − 34.53 to − 57.34 kcal/mol. Amorphastilbol (ZINC5158604) showed the highest binding energy of − 57.34 kcal/mol, followed by mangiferin of − 52.65 kcal/mol which is tabulated in Table 2. For LRP6 protein, mangiferin showed the highest binding free energy of − 44.19 kcal/mol, and the least energy was seen for Rosamarinic acid of − 0.33 kcal/mol. For DKK1 protein, ginsenoside had the highest binding energy of − 68.01 kcal/mol followed by mangiferin with the binding energy of − 48.53 kcal/mol, and the least binding energy was for scutellarein of − 22.25 kcal/mol. Similarly, for GSK-3β protein the highest binding energy was shown by curcumin, and the least was seen for mangiferin with the binding energy of − 34.91 kcal/mol. Finally, the binding energy calculation for WIF1 protein showed the highest binding energy for Curcumin (− 71.13 kcal/mol) and least for morin (− 34.70 kcal/mol). The details of the binding energy of the top 2 hit molecules for each protein are listed in Table 2. Analysis of the binding energy for all the top hit molecules for different proteins revealed that mangiferin showed the highest binding energy for LRP6 and lowest for GSK-3β. However, it showed the highest dock score so, for further confirmation of the binding and stability of mangiferin we have performed an MD simulation study for mangiferin complex with all the selected proteins in this current study.

#### Drug like property of top hit molecules

The success and discovery of a new drug not only depend on its target specificity and selectivity but also depends on pharmacokinetic properties. The selected hit molecules for different target proteins were analyzed using the QikProp module of Schrodinger suite. The selected hit molecules molecular weight was between 130.0 and 725.0. QPlogS is the predicted aqueous solubility in mol dm-3 all the compounds except nicotine have values within the acceptable range of − 6.5–0.5. QPlogPo/w predicted octanol/ water partition coefficient which is acceptable between − 2.0–+ 6.5 all compounds fall well within the acceptable arrange showing hydrophobic and lipophilic balance which is essential for the drug to be absorbed by the body and reach to the target site. QPPCaco stands for predicted Caco-2 cell permeability which signifies gut blood barrier permeability only 5 compounds tridolgosir, butein, scutellarein, curcumin, and resveratrol were in the acceptable range. All the molecules satisfied Lipinski rule of five. QPlogHERG is the predicted IC50 value for blockage of HERG K + channel signifying the potential of the compound to show toxicity. Most of the hit molecules have shown acceptable value for HERG toxicity except ZINC5158604, ZINC100067274, and ZINC103539689 molecules. Mangiferin has an experimental Log P value of + 2.73 [48] that supports its blood–brain permeability. All the predicted pharmacokinetic properties of the top hit molecules are tabulated in (Supplementary Table 1).

#### MD simulation

MD simulation is considered a fundamental computational tool for analyzing dynamic events of ligand–protein complexes. MD simulation has several advantages over XP-docking. It overcomes the problem of the rigid nature of protein in normal XP-docking. In MD simulation, the protein–ligand complex is in dynamic nature, and it also allows conformational changes of ligands within the active site of a protein. This phenomenon also mimics the scenario of a biological system, where the stability of the protein–ligand complex is evaluated in the simulated water boundary. Considering docking score, non-bonding interactions with vital amino acid residues, and binding energy, the top two hits were identified and selected for MD simulation with 2YGO, 3S2K Chain A (LRP6), 3S2K Chain X (DKK1), 1Q5K, and 4M0F proteins. In the current study, an attempt to evaluate its binding potential with other modulators of Wnt signaling and its possible role in AD has been made.

**Fig. 2:**
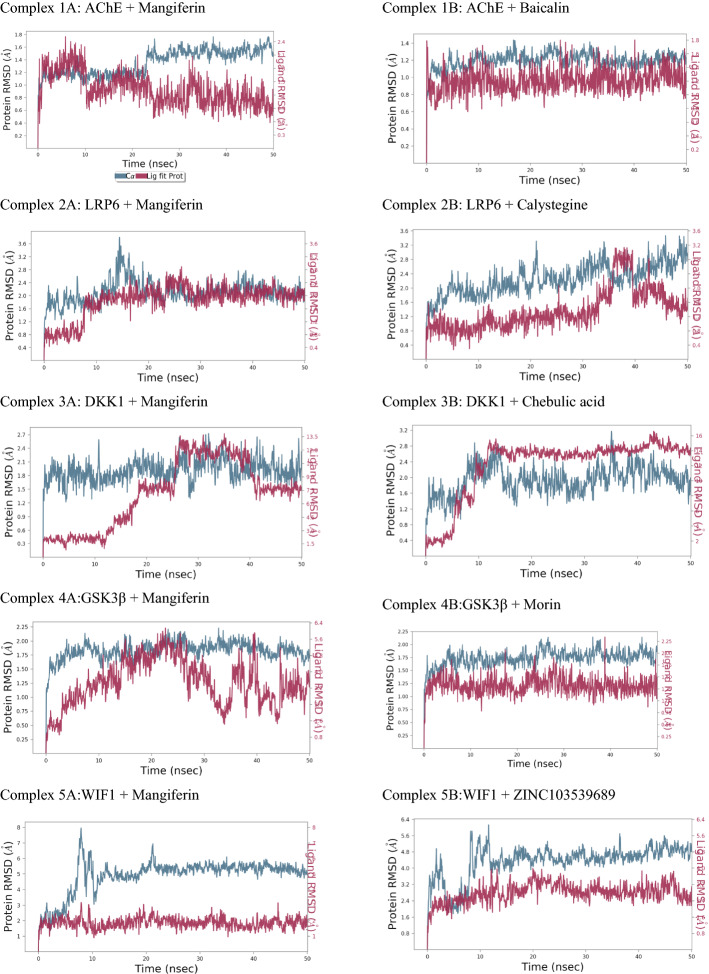
Root mean square deviation plot of top hit ligands with different proteins (AChE, LRP6, DKK1, GSK-3β, and WIF1)

**Fig. 3:**
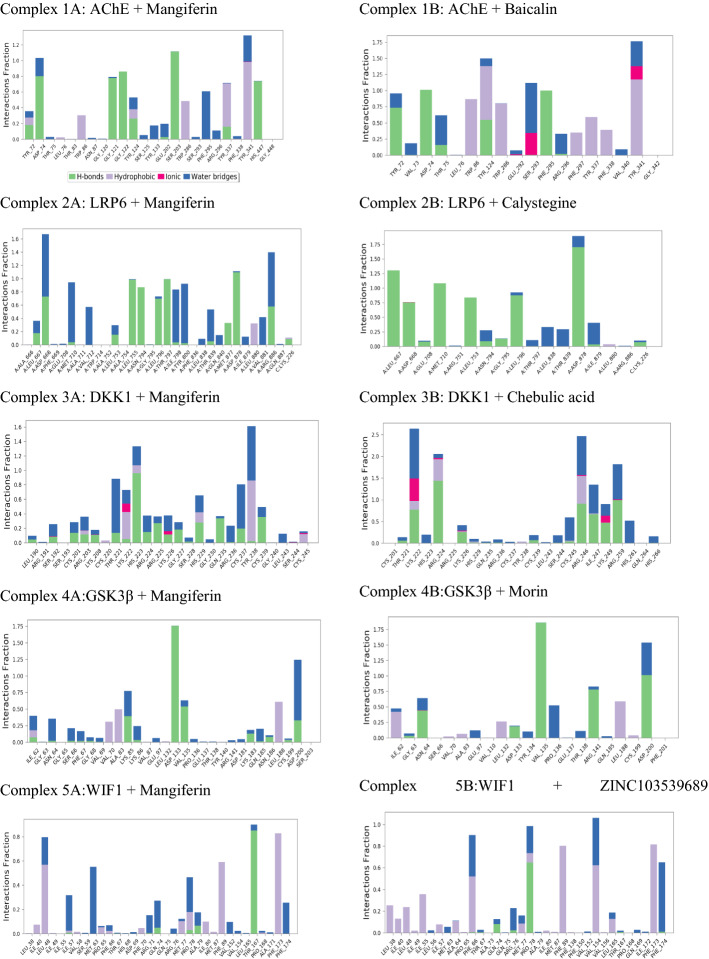
Protein–ligand contact of top hit ligands with different proteins (AChE, LRP6, DKK1, GSK-3β, and WIF1)

The frame was taken every 50 ps for the simulation period of 50 ns which resulted in 1000 frame generation and was saved in trajectory. Based on results obtained from MD simulation, a simulation interaction diagram was generated with root mean square deviation (RMSD), and a Protein–Ligand interaction plot was computed for 2YGO, 3S2K, 1QSK, and 4M0F proteins with the ligand mangiferin and 2^nd^ top hit ligand Figs. 2, 3. The RMSD and Ligand fit Plot help in estimating the ligand–protein complex stability. In the current study, MD simulation of mangiferin ligand and 2nd hit molecule from docking study was performed for five ligand–protein complexes viz., Complex 1A: AChE + Mangiferin, Complex 1B: AChE + Baicalin; Complex 2A: LRP6 + Mangiferin, Complex 2B: LRP6 + Calystegine; Complex 3A: DKK1 + mangiferin, Complex 3B: DKK1 + Chebulic acid; Complex 4A: GSK3β + mangiferin, Complex 4B: GSK3β + Morin; and Complex 5A:WIF1 + Mangiferin, Complex 5B:WIF1 + ZINC103539689.

#### Analysis of MD simulation for top hit molecules for AChE protein

The complex 1A was initially stable for 0–24 ns, but the further drift was observed for 24–-50 ns. Average RMSD values for AChE and mangiferin were found to be 2.4 Å and 0.9 Å, respectively. RMSD values of both protein and ligand were found to be within range for complex 1A. In complex 1A, polar interactions with SER203 and HIS447 were retained and lost with THR75. New hydrophobic bond interaction was observed with PHE295, retained with TYR337 and TYR341, and lost with other amino acids as compared to XP docking. The charged negative interactions were retained and were lost with ASP74 and GLU202. New π–π stacking interaction was observed with TYR341 and lost with TRP286. H-bond interactions with SER203 and HIS447 were retained and were lost with TYR72. New H-bond interactions were found with ASP74, PHE295, and GLY122. Complex 1B was stable throughout the simulation of 50 ns RMSD of both protein and ligand which was less than 2 Å. Hydrogen bond interactions like PHE295, ASP74 which were seen in the XP docking pose was retained, and new bonding ASP72 was formed during MD simulation. TYR341 which showed Hydrogen interaction in XP docking pose was changed to a π–π type of interaction in MD. New π–π type of interaction with TRP86, TYR337, and TYR124 was seen during MD simulation.

In acetylcholine esterase amino acids TRP86, TYP286 and TYR341, SER293, TYR72, and TYR124 play a critical role in the inhibition of enzyme [28]. In our study, Complex 1A showed interaction with only TYR341 in both XP docking and MD simulation. Complex 2B showed interaction with TRP286, TYR341 both in XP docking and MD simulation, and new interaction with TYR72, TYR124 was formed in MD simulation which is both essential for AChE inhibition. From this, we get to know that complex 2B interacts with key residue; therefore, baicalin has more potential to inhibit to AChE enzyme than mangiferin.

#### Analysis of MD simulation for top hit molecules for LRP6 protein

In complex 2A, initial drift was observed for 0–8 ns; eventually, ligand–protein complex got stabilized for the simulation period of 50 ns. Additionally, in complex 2A, a drift was observed in between for 13–17 ns. The RMSD value of protein and ligand for complex 2A was observed to be 2 Å within an acceptable range (1–3 Å). H-bond interactions were retained with ASP668, LEU755, TYR480, MET710, and THR797, which showed new interactions with ALA 881, ARG886, VAL712, LEU796, ASP878, MET877, ASN794, ILE798, and THR839, and lost with GLU708 and LEU573 in complex 2. These new H-bond interactions may contribute to the additional stability of complex 2A. The new water bridge interactions were found with amino acids VAL881, ARG886, VAL712, TYR800, MET710, ILE798, THR839, and ASP668, as compared to XP docked poses. The hydrophobic interactions were found with VAL881, retained with MET710, VAL712, LEU755, LEU796, TYR800, MET877, LEU880, and ILE798, and lost with other amino acids. The polar interactions were retained with ASN794, THR797, THR839 and lost with GLU840 and GLU887. The charged positive interaction with ARG886 was retained in MD simulation. The new charged negative interaction was found with GLU708 by retaining other interactions. Several in vivo studies have proved the benefit of mangiferin in memory improvement based on the anti-inflammatory and acetylcholinesterase activity [49, 50]. Complex 2B was stable from 0 to 35 ns later after 35 ns fluctuation in protein and ligand was seen. Hydrogen bond interaction seen in XP docking with MET710, ASP878, LEU667 was retained in MD simulation. Interaction with ASP838, LEU753 which was seen in XP docking was not seen in MD but new interaction with LEU796, ASP668 was formed in MD simulation.

After comparing the RMSD plot for both the ligands, we could conclude that the RMSD plot for the mangiferin–LRP6 complex was more stable with less fluctuation indicating the strong and good binding throughout the simulation duration. In LRP6 interactions with amino acids GLU663, ILE681, TYR708, ASP748, SER749, ARG751, TRP767, GLY769, ARG792, ASN794, LEU810, ASP811, ASP830, TRP850, and MET877 are important. However, the occurrence of the interactions with these residues was missing for both ligands in the current MD simulation.

#### Analysis of MD simulation for top hit molecules for DKK1 protein

Complex 3A was stable for 22–28 ns, 31–36 ns, 38–41 ns, and 46–49 ns, and drift was observed for 0-–22 ns, 28–31 ns, 36–38 ns, 41–46 ns, and 49–50 ns. The RMSD values were found to be 10.5 Å and 7.5 Å, for protein and ligand, respectively. In complex 3A, polar interactions were retained with HIS223 and THR221 and lost with HIS229, SER192, and GLU235. Formation of new H-bonds was observed with HIS223, CYS237, and CYS239, retained with THR221 and lost with SER192, CYS201, LYS208, and HIS229. Hydrophobic interactions were retained with TYR238 and CYS237, whereas lost with other amino acids. New hydrophobic interaction was observed with CYS239 and water bridge-type interaction with CYS237 and CYS239. The charged positive interaction was lost with ARG191, ARG203, LYS208, LYS222, and ARG236, as compared to XP ligand interactions. Complex 3B was stable for 5–15 ns and drift was observed for 0–5 ns and 15–50 ns. Protein and ligand showed RMSD values of 14 Å and 10 Å, respectively. During MD simulation, new hydrophobic and water bridge types of interactions were formed with ILE247 and lost other hydrophobic interactions as compared to XP docking. Similarly, new polar interaction was formed with HIS261 and lost other polar interactions. New π–π cation interactions were observed with ARG246 and ARG224 as compared to XP ligand docking interactions. Charged negative interaction was lost with ARG236, and newly charged negative interactions were observed with LYS249, ARG224, ARG259, LYS222, and ARG246. Additionally, water bridge type of interactions was observed with amino acid residues ARG259, HIS261, LYS222, and ILE247. The salt bridge type of interaction with LYS222 is retained in MD simulation as compared to XP ligand docking interactions.

Based on previous reports, amino residues CYS217-ARG237 and CYS233-CYS253 compromise the cysteine-rich domain and it is important for inhibition of Wnt signaling pathway [45]. From this region in complex 3B, HIS223, THR221, CYS237, THR238, and THR239 were present in MD simulation. Interaction with other amino acids such as GLN235, ARG236, CYS245, CYS220, LYS222, and LYS229 was lost in ligand interactions of MD simulation as compared to XP docking. In complex 3B, amino acid residues ARG2224, LYS222, LYS249, ARG246, and ILE247 were present in MD simulation, whereas few amino acid residues such as CYS220, ARG236, GLN235, HIS229, HIS223, LYS222, THR221, CYS237, TYR238, LYS239, and LYS245 were lost in MD simulation as compared to XP docking. In our study, complex 3A showed interaction with THR221 in both ligand interactions of XP docking and MD simulation, whereas LYS222 and ARG236 were only present in XP docking and lost in MD simulation. In complex 3B, amino acid LYS222 was present in ligand interactions of XP docking and MD simulation, whereas ARG224 and ARG259 were only found in ligand interactions of MD simulation. Therefore, finding from this study indicates complex 3B is more stable as compared to complex 3A, as most of the crucial amino acids are present in complex 3B.

#### Analysis of MD simulation for top hit molecules for GSK-3β protein

In complex 4A, the initial fluctuation was observed for 0–15 ns, the complex got stabilized for 15–27 ns, and further drift was observed for 27–50 ns. In complex 4A, H-bond interaction was retained with ASP133 and lost with LYS183, ASN186, and ASP200. The new H-bond interaction was found with VAL135 as compared to XP docking. Hydrophobic interactions were retained with VAL70, ALA83, LEU188, and VAL135 and lost with other amino acids. All charged positive interactions were lost in MD simulation as compared to XP docking. Charged negative interactions were lost with ASP181 and ASP200 and retained with ASP133. Complex 4B possesses the RMSD value 2 Å and 1.50 Å. In complex 4B, the difference between RMSD values was not more than 3 Å, which indicates complex 4B is stable. As compared to XP docking, the hydrophobic bond interactions were retained with LEU188, PRO136, VAL135, and ILE62 and lost with CYS199, VAL110, ALA83, LEU132, TYR134, and VAL70. Amino acid residues PRO136 and VAL135 also showed water bridge type and hydrogen bond interactions. The charged positive interactions with ASP200 are retained, and new hydrogen bond and water bridge type of interactions were observed with ASP200 as compared to XP ligand docking interactions. Polar interaction was lost with THR138 as compared to XP docking, and new polar and hydrogen bond interactions were observed with ASN64. Similarly charged negative interaction was retained with ARG141in MD simulation, and amino acid residue ARG141 also showed hydrogen bond interaction as compared to XP docking.

In GSK-3β protein, the co-crystallized inhibitor showed that the inhibitor occupies the hinge region along with ATP pocket through the formation of three hydrogen bonds with residue VAL135 [46]. In our study, complex 4A showed interactions with VAL135 and LEU188 in ligand interactions of MD simulation as compared to XP docking. Similarly, for complex 4B the interactions with the key residues VAL135, LEU188, and ASP200 were found to be retained in MD simulation. Additionally, the RMSD plot was more stable with fewer fluctuations for the complex 4B in comparison to that of complex 4A.

#### Analysis of MD simulation for top hit molecules for WIF1 protein

RMSD plot of different proteins and mangiferin is shown in Fig. 2. The complex 5A was found to be stable for 6 ns and a later drift was noted for 6–50 ns in the ligand–protein complex. The RMSD value of protein was found to be 5.2 Å and ligand was 2.0 Å. During 50 ns MD simulation of complex 5A, polar interaction with THR167 was retained as compared to XP ligand–protein interactions. H-bond interactions were retained with THR167 and lost with PRO078. Similarly, π–π interactions with PHE173 were retained and lost with PHE89. Hydrophobic bond interactions were retained with PHE89, MET63, and LEU48 and lost with other amino acids. Additionally, hydrogen and water bridge interactions were observed with MET63. For complex 5B initially, the protein was not stable till 15 ns after that it got stabilized till the end of the simulation. The ligand RMSD was less than 4 Å throughout the simulation showing that it has not undergone much change throughout the simulation. Hydrogen bond interaction with PHE173 was retained in both XP docking and MD simulation. New interactions with PRO173, PHE174, PHE66, and VAL154 were seen in MD simulations. In WIF1 protein, MET77 is an important amino acid residue. The involvement of both WIF domain (38–177 amino acid residue) and EGF domain has been noted in the binding of Wnt proteins. In our study, both in complex 5A and complex 5B interactions with MET77 were not seen. However, the RMSD plot for complex 5B showed less deviation in RMSD between ligand and protein during the simulation in comparison with the 5A complex Fig. 3.

## Conclusion

Wnt signaling plays an important role in the cell growth, proliferation, embryogenesis, and organogenesis process. Phytochemical database was considered for screening their potential to modulate markers of the Wnt signaling pathway. Different proteins (viz. LRP6, DKK1, WIF1, GSK-3β) linked with Wnt signaling pathway and AChE have been selected to explore the binding affinity, interactions of the selected phytomolecules and virtually evaluate their potential to upregulate Wnt signaling. The inbound ligand was considered for the generation of the grid for docking simulation in the case of proteins GSK-3β and AChE, whereas a possible drug-binding pocket predicted by the sitemap tool was chosen for grid generation for the proteins LRP6, DKK1, and WIF1. Based on the computational molecular docking, the top five molecules were selected for each target protein. The top two molecules were chosen for further analysis of their binding and stability using MD simulation. In all the proteins, mangiferin was found to have the highest docking as well as binding interactions with the key amino acid residues. For AChE protein, although mangiferin has shown a high dock score of − 14.205 kcal/mol with the xanthone ring forming π–π stacking interaction with key interacting residue TRP286, the 2nd hit molecule baicalin with a dock score of − 13.224 kcal/mol has shown a stable RMSD throughout the simulation and exhibited π–π stacking interactions with TRP286 and TRR341 during entire MD simulation period. This shows the possibility of Baicalin to be a potent molecule with strong and stable binding than mangiferin for AChE protein. With DKK1 protein, mangiferin has shown the highest dock score of − 11.155 kcal/mol, but chebulic acid has shown stable interactions in MD simulations with important interactions with key residues ARG224, LYS222, and ARG259. With WIF1 protein, the deviation in the RMSD of ligand and the protein was higher for mangiferin and lower for ZINC103539689 indicating more stable binding in MD simulation for ZINC103539689. For GSK-3β protein morin, with a dock score of − 9.42 kcal/mol has shown stable RMSD throughout the MD simulation as compared to mangiferin although it had a dock score of − 10.344 kcal/mol. In the case of LRP6 protein, mangiferin has shown the highest docking score as well as stable RMSD and interactions with the key residues during MD simulation in comparison to the 2nd hit molecule calystegine. From this in silico-based study, we report that mangiferin could be a potential molecule targeting Wnt signaling pathway modulating the LRP6 activity, baicalin for AChE activity, chebulic acid for DKK1, ZINC103539689 for WIF1, and morin for GSk-3β protein. However, further validation of the activity is warranted based on in vivo and in vitro experiments for better understanding and strong claim.

## Supplementary Information

Below is the link to the electronic supplementary material.

Supplementary file1 (DOCX 1591 KB)

## References

[1] Nusse R, Brown A, Papkoff J (1991). A new nomenclature for int-1 and related genes: the Wnt gene family. Cell.

[2] Willert K, Nusse R (2012). Wnt proteins. Cold Spring Harb Perspect Biol.

[3] Inestrosa NC, Toledo EM (2008). The role of wnt signaling in neuronal dysfunction in Alzheimer’s Disease. Mol Neurodegener.

[4] Ciani L, Salinas PC (2005). WNTS in the vertebrate nervous system: from patterning to neuronal connectivity: signalling in neural development. Nat Rev Neurosci.

[5] Lie DC, Song H, Colamarino SA (2004). Neurogenesis in the adult brain: new strategies for central nervous system diseases. Annu Rev Pharmacol Toxicol.

[6] Lie DC, Colamarino SA, Song HJ (2005). Wnt signalling regulates adult hippocampal neurogenesis. Nature.

[7] Wang L, Liu Y, Li S (2015). Wnt signaling pathway participates in valproic acid-induced neuronal differentiation of neural stem cells. Int J Clin Exp Pathol.

[8] Lange C, Mix E, Rateitschak K, Rolfs A (2006). Wnt signal pathways and neural stem cell differentiation. Neurodegener Dis.

[9] Caraci F, Busceti C, Biagioni F (2008). The Wnt antagonist, Dickkopf-1, as a target for the treatment of neurodegenerative disorders. Neurochem Res.

[10] Semënov MV, Zhang X, He X (2008). DKK1 antagonizes Wnt signaling without promotion of LRP6 internalization and degradation. J Biol Chem.

[11] Gordon MD, Nusse R (2006). Wnt signaling: multiple pathways, multiple receptors, and multiple transcription factors. J Biol Chem.

[12] Caricasole A, Copani A, Caruso A (2003). The wnt pathway, cell-cycle activation and β-amyloid: novel therapeutic strategies in Alzheimer’s disease?. Trends Pharmacol Sci.

[13] Lim FT, Ogawa S, Smith AI, Parhar IS (2017). Proteomics identification of potential candidates involved in cell proliferation for early stage of brain regeneration in the adult zebrafish. Zebrafish.

[14] Folke J, Pakkenberg B, Brudek T (2019). Impaired wnt signaling in the prefrontal cortex of Alzheimer’s disease. Mol Neurobiol.

[15] Caricasole A, Copani A, Caraci F (2004). Induction of Dickkopf-1, a negative modulator of the Wnt pathway, is associated with neuronal degeneration in Alzheimer’s brain. J Neurosci.

[16] Alvarez AR, Godoy JA, Mullendorff K (2004). Wnt-3a overcomes β-amyloid toxicity in rat hippocampal neurons. Exp Cell Res.

[17] Bourhis E, Tam C, Franke Y (2010). Reconstitution of a frizzled8·Wnt3a·LRP6 signaling complex reveals multiple Wnt and Dkk1 binding sites on LRP6. J Biol Chem.

[18] Rismani E, Rahimi H, Arab SS (2018). Computationally design of inhibitory peptides against wnt signaling pathway. In silico insight on complex of DKK1 and LRP6. Int J Pept Res Ther.

[19] Brogi S, Maramai S, Brindisi M (2017). Activation of the wnt pathway by small peptides: rational design, synthesis and biological evaluation. ChemMedChem.

[20] Sankhe R, Rathi E, Manandhar S (2021). Repurposing of existing FDA approved drugs for Neprilysin inhibition: an in-silico study. J Mol Struct.

[21] Thomford N, Senthebane D, Rowe A (2018). Natural products for drug discovery in the 21st century: innovations for novel drug discovery. Int J Mol Sci.

[22] Tripathi K (2009). Curcumin-the spice of life-II. Res J Pharmacogn Phytochem.

[23] Santana de Oliveira M, Neves da Cruz J, Almeida da Costa W (2020). Chemical composition, antimicrobial properties of siparuna guianensis essential oil and a molecular docking and dynamics molecular study of its major chemical constituent. Molecules.

[24] da Silva Júnior OS, de Franco CJP, de Moraes AAB (2021). In silico analyses of toxicity of the major constituents of essential oils from two Ipomoea L. species. Toxicon.

[25] Malinauskas T, Aricescu AR, Lu W (2011). Modular mechanism of Wnt signaling inhibition by Wnt inhibitory factor 1. Nat Struct Mol Biol.

[26] Ahn VE, Chu MLH, Choi HJ (2011). Structural basis of Wnt signaling inhibition by dickkopf binding to LRP5/6. Dev Cell.

[27] Bhat R, Xue Y, Berg S (2003). Structural insights and biological effects of glycogen synthase kinase 3-specific inhibitor AR-A014418. J Biol Chem.

[28] Cheung J, Gary EN, Shiomi K, Rosenberry TL (2013). Structures of human acetylcholinesterase bound to dihydrotanshinone i and territrem B show peripheral site flexibility. ACS Med Chem Lett.

[29] PH R,  (2013). Amyloid beta-induced glycogen synthase kinase 3β phosphorylated VDAC1 in Alzheimer’s disease: implications for synaptic dysfunction and neuronal damage. Biochim Biophys Acta.

[30] Liu H, Luo K, Luo D (2018). Guanosine monophosphate reductase 1 is a potential therapeutic target for Alzheimer’s disease. Sci Rep.

[31] Friesner RA, Banks JL, Murphy RB (2004). Glide: a new approach for rapid, accurate docking and scoring. 1. Method and assessment of docking accuracy. J Med Chem.

[32] Halgren TA (2009). Identifying and characterizing binding sites and assessing druggability. J Chem Inf Model.

[33] Irwin JJ, Shoichet BK (2005). ZINC–a free database of commercially available compounds for virtual screening. J Chem Inf Model.

[34] Manasa B, Manandhar S, Hari G (2021). Virtual structure-based docking, WaterMap, and molecular dynamics guided identification of the potential natural compounds as inhibitors of protein-tyrosine phosphatase 1B. J Mol Struct.

[35] Genheden S, Ryde U (2015). The MM/PBSA and MM/GBSA methods to estimate ligand-binding affinities. Expert Opin Drug Discov.

[36] Santos CBR, Santos KLB, Cruz JN (2021). Molecular modeling approaches of selective adenosine receptor type 2A agonists as potential anti-inflammatory drugs. J Biomol Struct Dyn.

[37] Araújo PHF, Ramos RS, da Cruz JN (2020). Identification of potential COX-2 inhibitors for the treatment of inflammatory diseases using molecular modeling approaches. Molecules.

[38] Li J, Abel R, Zhu K (2011). The VSGB 2.0 model: a next generation energy model for high resolution protein structure modeling. Proteins Struct Funct Bioinform.

[39] Ioakimidis L, Thoukydidis L, Mirza A (2008). Benchmarking the reliability of QikProp. Correlation between experimental and predicted values. QSAR Comb Sci.

[40] Cheung J, Rudolph MJ, Burshteyn F (2012). Structures of human acetylcholinesterase in complex with pharmacologically important ligands. J Med Chem.

[41] Cheng Z, Biechele T, Wei Z (2011). Crystal structures of the extracellular domain of LRP6 and its complex with DKK1. Nat Struct Mol Biol.

[42] Bao J, Zheng JJ, Wu D (2012). The structural basis of DKK-mediated inhibition of Wnt/LRP signaling. Sci Signal.

[43] Chen S, Bubeck D, MacDonald BT (2011). Structural and functional studies of LRP6 ectodomain reveal a platform for Wnt signaling. Dev Cell.

[44] Ren C, Gu X, Li H (2019). The role of DKK1 in Alzheimer’s disease: a potential intervention point of brain damage prevention?. Pharmacol Res.

[45] Gregory CA, Perry AS, Reyes E (2005). Dkk-1-derived synthetic peptides and lithium chloride for the control and recovery of adult stem cells from bone marrow. J Biol Chem.

[46] Palomo V, Soteras I, Perez DI (2011). Exploring the binding sites of glycogen synthase kinase 3. identification and characterization of allosteric modulation cavities. J Med Chem.

[47] Pandey MK, DeGrado TR (2016). Glycogen synthase kinase-3 (GSK-3)-targeted therapy and imaging. Theranostics.

[48] Ochocka R, Hering A, Stefanowicz-Hajduk J (2017). The effect of mangiferin on skin: penetration, permeation and inhibition of ECM enzymes. PLoS ONE.

[49] Kasbe P, Jangra A, Lahkar M (2015). Mangiferin ameliorates aluminium chloride-induced cognitive dysfunction via alleviation of hippocampal oxido-nitrosative stress, proinflammatory cytokines and acetylcholinesterase level. J Trace Elem Med Biol.

[50] Biradar SM, Joshi H, Chheda TK (2012). Neuropharmacological effect of Mangiferin on brain cholinesterase and brain biogenic amines in the management of Alzheimer’s disease. Eur J Pharmacol.

