# Supplementary material for: Molecular dynamics and structure-based virtual screening and identification of natural compounds as Wnt signaling modulators: possible therapeutics for Alzheimer’s disease
Source: Mol Divers. 2022 Feb 11;26(5):2793–811. doi: 10.1007/s11030-022-10395-8 (PMC9532339; doi:10.1007/s11030-022-10395-8)
Supplement: Supplementary file 1 — Supplementary file1 (DOCX 1591 KB) [file 11030_2022_10395_MOESM1_ESM.docx]

**Table 1:** Predicted pharmacokinetic properties of the selected hit molecules using QikProp module.

|  | **CNS** | **Mol wt.** | **QPlogPo/w** | **QPlogS** | **QPlogHERG** | **QPPCaco** | **QPlogBB** | **Percent Human Oral Absorption** | **Rule of Five** |
| --- | --- | --- | --- | --- | --- | --- | --- | --- | --- |
| Recommended values | -2.0 - +2.0 | 130.0-725.0 | -2.0 - +6.5 | -6.5-0.5 | Concern Below -5 | <25 poor, >500 great | -3.0 - +1.2 | >80% is high  <25% is poor | Max-4 |
| ZINC33832403(Mangiferin) | -2 | 422.345 | -1.753 | -2.483 | -5.091 | 3.841 | -3.471 | 1.223 | 2 |
| ZINC3943903 (Baicalin) | -2 | 446.367 | 0.4 | -3.703 | -4.167 | 1.112 | -3.553 | 4.198 | 2 |
| ZINC899870 (Rosmarinic acid) | -2 | 360.32 | 1.145 | -3.23 | -4.085 | 2.275 | -3.398 | 40.041 | 0 |
| ZINC5158604 (Amorphastilbol) | -1 | 348.484 | 6.166 | -7.018 | -6.582 | 1756.705 | -0.905 | 100 | 1 |
| ZINC3881558 (Morin) | -2 | 302.24 | 0.393 | -2.796 | -4.996 | 22.082 | -2.292 | 53.3 | 0 |
| ZINC12504453 (Calystegine) | -1 | 175.184 | -1.817 | 0.238 | -3.222 | 33.917 | -0.76 | 43.698 | 0 |
| ZINC3875041 (Tridolgosir) | -1 | 173.211 | -1.082 | 0.082 | -3.33 | 143.082 | -0.216 | 59.192 | 0 |
| ZINC19968 (Escosyl) | -2 | 340.286 | -1.628 | -1.9 | -4.745 | 19.177 | -2.58 | 40.372 | 0 |
| ZINC13385490 (Chebulic acid) | -2 | 356.242 | -1.121 | -1.622 | 1.997 | 0.011 | -3.566 | 0 | 2 |
| ZINC12428433 (Butein) | -2 | 272.257 | 1.247 | -2.741 | -5.243 | 48.349 | -2.192 | 64.393 | 0 |
| ZINC5842416 (Scutellarein) | -2 | 286.24 | 0.956 | -2.967 | -4.963 | 51.388 | -1.835 | 63.167 | 0 |
| ZINC100067274 (Curcumin) | -2 | 368.385 | 2.677 | -4.295 | -6.187 | 161.689 | -2.186 | 82.151 | 0 |
| ZINC1536 (Honokiol) | 0 | 266.339 | 4.969 | -4.248 | -5.641 | 1565.77 | -0.683 | 100 | 0 |
| ZINC103539689 | -2 | 482.443 | 1.715 | -5.072 | -6.194 | 19.648 | -2.855 | 60.133 | 0 |
| ZINC84669328 (Claulansine F) | 0 | 307.348 | 3.641 | -5.032 | -5.082 | 1301.452 | -0.422 | 100 | 0 |
| ZINC967597 (Thymol) | 1 | 150.22 | 3.298 | -2.323 | -3.574 | 3816.681 | 0.086 | 100 | 0 |
| ZINC2392265 (Isohumulone) | -2 | 362.465 | 3.792 | -4.929 | -4.679 | 989.736 | -1.107 | 100 | 0 |
| ZINC6787 (Resveratrol) | -2 | 228.247 | 1.987 | -2.766 | -5.303 | 276.799 | -1.29 | 82.287 | 0 |
| ZINC391812 (Nicotine) | 2 | 162.234 | 1.187 | 0.758 | -4.417 | 1274.844 | 0.718 | 89.479 | 0 |
| ZINC95617916 (Avenanthramide A ) | -2 | 299.282 | 2.016 | -3.417 | -3.965 | 15.016 | -2.142 | 59.805 | 0 |

**Table 2:** Dock score, binding energy, and interacting residues for Acetylcholinesterase enzyme (PDB id: 4M0F) of top five molecules.

| **Molecular docking in XP docking mode (PDB id: 4M0F)** | | | | |
| --- | --- | --- | --- | --- |
| **Compound** | **XP Dock score (kcal/mol)** | **MMGBSA dG Bind (kcal/mol)** | **Non-bonding interactions** | **2D Interaction diagram** |
| ZINC33832403 (Mangiferin) | -14.205 | -52.65 | **H-Bond:** TYR72, SER203, HIS447  **π-π interaction:** TRP286  **Hydrophobic:** TYR72, LEU76, TRP86, TYR124, ALA204, TYR286, PHE297, TYR337, PHE338, TYR341  **Polar:** THR75, HIS447, SER203  **Charged Negative**: ASP74, GLH202 | 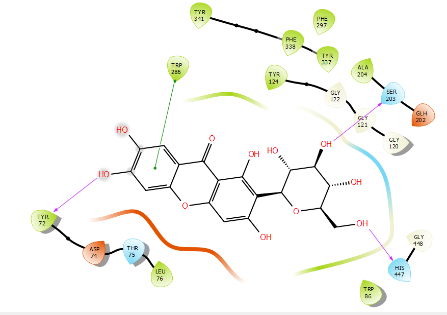 |
| ZINC3943903  (Baicalin) | -13.224 | -43.28 | **H-Bond:** ASP74, PHE295, TYR341  **π-π interaction:** TRP286  **Hydrophobic:** TYR72, LEU76, TRP86, TYR124, TRP286, VAL294, PHE295, PHE297, TYR337, PHE338, TYR341  **Polar:** THR75, SER293, HIS447  **Charged Negative:** ASP74 | 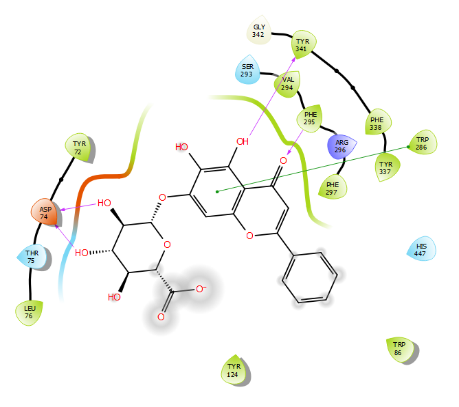 |
| ZINC899870 (Rosmarinic acid) | -11.483 | -34.53 | **H-Bond:** ASP74, GLY122, **SER203,** ARG296  **π-π interaction:** TRP286, **HIS447**  **Hydrophobic:** TYR72, LEU76, TRP86, TYR124, ALA204, TRP286, VAL294, PHE295, PHE297, TYR337, PHE338, TYR341  **Polar:** THR75, SER203, HIS447  **Charged Negative:** ASP74, GLH202 | 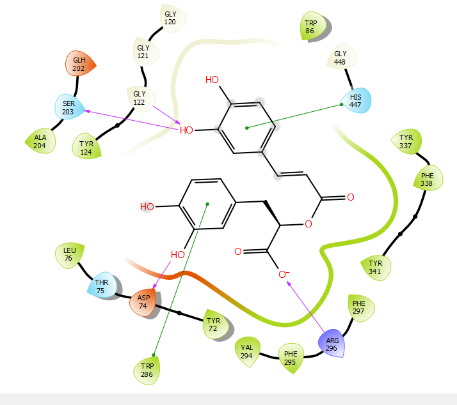 |
| ZINC5158604 (Amorphastilbol) | -10.8 | -57.34 | **H-Bond:** PHE295  **Hydrophobic:** TRP86, TYR124, TRP286, LEU289, VAL294, PHE295, PHE297, TYR337, PHE338, TYR341  **Polar:** SER203, HIE287, GLN291, SER293, HIS447  **Charged Negative:** GLU292  **Charged Positive:** ARG296 | 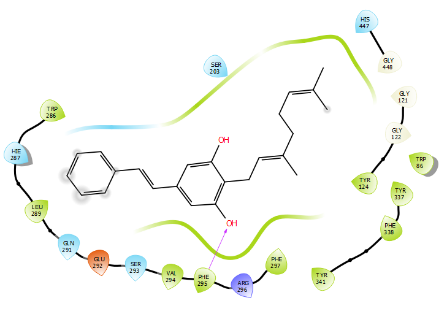 |
| ZINC3881558  (Morin) | -10.987 | -43.28 | **H-Bond:** ASP74, PHE295, ARG296  **π-π interaction :** TRP286, TYR341  **Hydrophobic:** TRP86, TYR124, LEU289, TRP286, VAL294, PHE295, PHE297, TYR337, PHE338, TYR341  **Polar:** THR83, SER293  **Charged Negative:** ASP74  **Charged Positive:**  ARG296 | 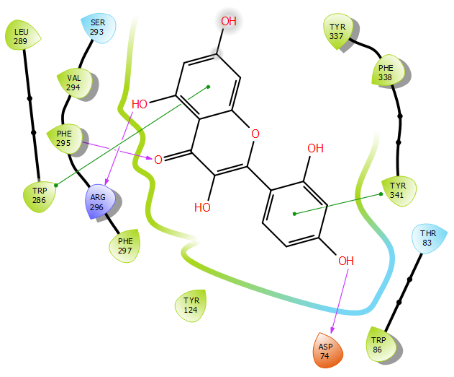 |

**Table 3:** Dock score, binding energy, and interacting residues for LRP6 protein (PDB id: 3S2K) of top five molecules.

| **Molecular docking in XP docking mode for LRP6 (PDB id: 3S2K)** | | | | |
| --- | --- | --- | --- | --- |
| **Compound** | **XP Dock score (Kcal/mol)** | **MMGBSA dG Bind (kcal/mol)** | **Non-bonding interactions** | **2D Interaction diagram** |
| ZINC33832403 (Mangiferin) | -10.960 | -44.19 | **H-Bond:** ASP668, GLU708, MET710, LEU753, LEU755, TYR800, THR797  **Hydrophobic:** PHE669, ALA666, MET710, ALA711, VAL712, TRP714, ALA752, LEU753, ALA754, LEU755, LEU796, ILE798, TYR800, LEU838, MET877, ILE879, LEU880  **Polar:** ASN794, THR797, THR839, GLN840, GLN887  **Charged Negative:** ASP668, GLU708, ASP878  **Charged Positive:** ARG886 | **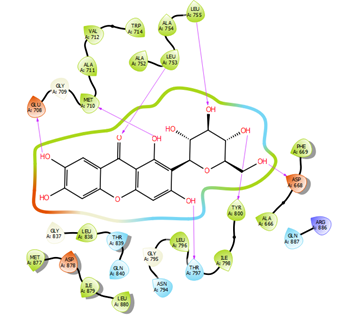** |
| ZINC12504453  (Calystegine) | -10.487 | -32.32 | **H-Bond:** LEU667, MET710, LEU753, LEU838, ASP878  **Salt Bridge:** ASP878  **Hydrophobic:** LEU667, ALA666, MET710, ALA711, ALA752, LEU753, ALA754, LEU796, LEU838, ILE879, LEU880  **Polar:** ASN794, THR797, THR839  **Charged Negative:** ASP668, ASP878 | **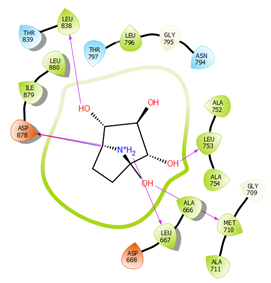** |
| ZINC3875041 (Tridolgosir) | -9.079 | -42.32 | **H-Bond:** MET710, LEU753, LEU796  **Salt Bridge:** ASP878  **Hydrophobic:** ALA666, LEU667, MET710, ALA711, ALA752, LEU753, ALA754, LEU796, LEU838, MEG877, ILE879, LEU880,  **Polar:** ASN794, THR797, THR839  **Charged Negative:** GLU708, ASP878 | 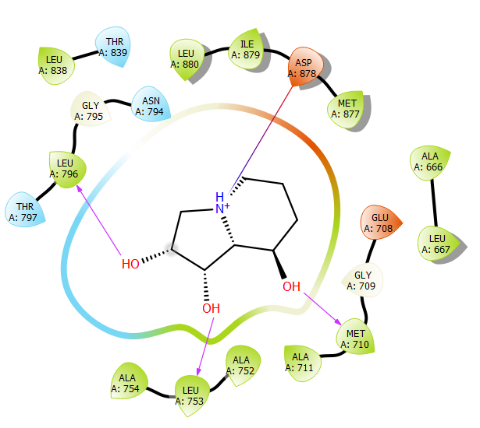 |
| ZINC19968  (Escosyl) | -9.049 | -22.38 | **H-Bond:** LEU753, LEU755, TYR800, LEU838, GLN887  **Hydrophobic:**  ALA666, LEU667, PHE669, MET710, ALA711, VAL712, TRP714, ALA752, LEU753, ALA754, LEU755, LEU796, ILE798, TYR800, LEU838, LEU880, ILE879  **Polar:** ASN794, THR797, THR839, GLN887  **Charged Negative:** ASP668, GLU708, ASP878  **Charged Positive:** ARG886 | 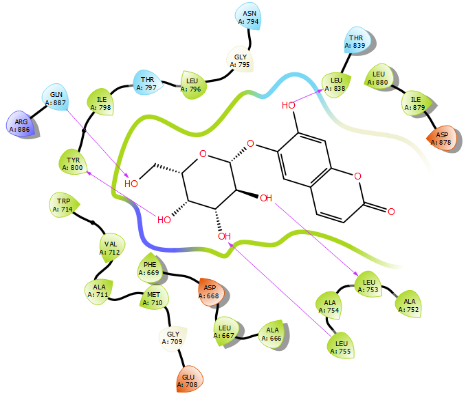 |
| ZINC899870 (Rosmarinic acid) | -8.913 | -0.33 | **H-Bond:** ASP668, GLU708, VAL712, ILE798, THR839, ASP878, ARG886, GLN887  **Salt Bridge**: ARG886  **Hydrophobic:**  ALA666, PHE669, MET710, ALA711, VAL712, TRP714, ALA752, LEU753, ALA754, LEU755, LEU796, ILE798, TYR800, LEU838, MET877, ILE879, LEU880  **Polar:** ASN794, THR797, THR839, GLN887  **Charged Negative:** ASP668, GLU708, ASP713, ASP878  **Charged Positive:** ARG886 | 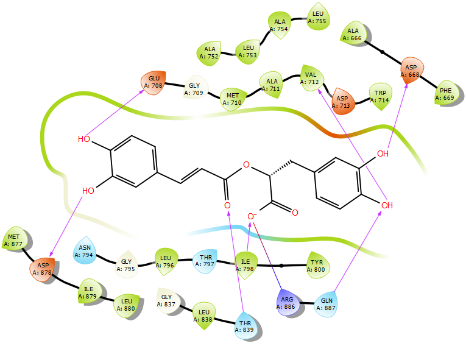 |

**Table 4:** Dock score, binding energy, and interacting residues for DKK1 protein (PDB id: 3S2K)of top five molecules

| **Molecular docking in XP docking mode for DKK1 (PDB id: 3S2K)** | | | | |
| --- | --- | --- | --- | --- |
| **Compound** | **XP Dock score (kcal/mol)** | **MMGBSA dG Bind (kcal/mol)** | **Non-bonding interactions** | **2D Interaction diagram** |
| ZINC33832403  (Mangiferin) | -11.155 | -48.53 | **H-Bond:** SER192, CYS201, LYS208, HIE229, THR221  **Hydrophobic:** LEU190, CYS200, CYS201, ALA202, CYS220, CYS237, TYR238, CYS245  **Polar:** SER192, THR221, HIS223, HIE229, GLN235  **Charged Positive:** ARG191, ARG203, LYS208, LYS222, ARG236 | 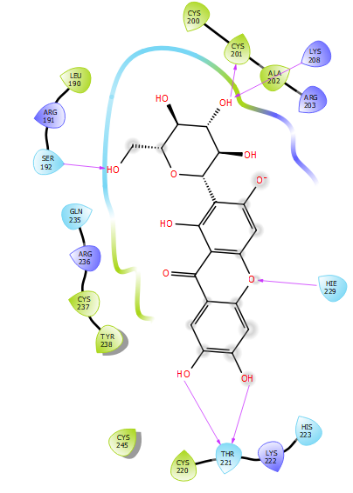 |
| ZINC000013385490 (Chebulic acid) | -9.972 | -34.28 | **H-Bond:** THR221, HIS223, HIE229, GLN235, ARG236, CYS239.  **Salt bridge:** LYS222  **Hydrophobic:** LEU214, CYS220, CYS237, TYR238, CYS239, CYS245  **Polar:** THR221, HIS223, HIE229, GLN235  **Charged Negative:** LYS222, ARG236 | 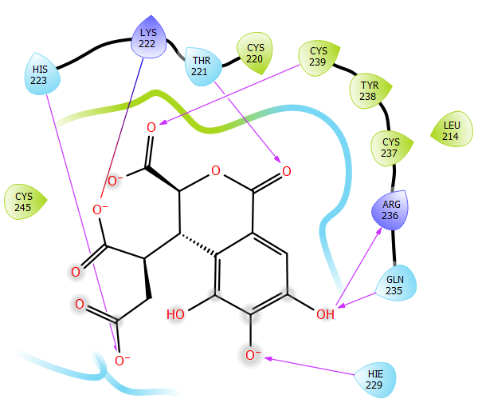 |
| Ginsenoside | -9.782 | -68.01 | **H-Bond:** CYS201, THR221, HIE229, GLN235, ARG236, CYS239, GLY242,  **Hydrophobic:** CYS200, CYS201, ALA202, CYS237, TYR238, CYS239, LEU243, CYS245  **Polar:** SER192, THR221, HIS223, HIE229, GLN235, SER244, HIE266  **Charged Negative:**  GLU241  **Charged Positive:**  LYS222, ARG236 | 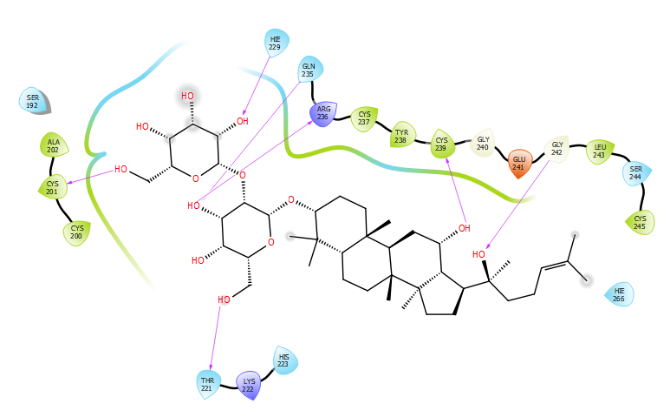 |
| ZINC000012428433 (Butein) | -9.460 | -26.14 | **H-Bond:** GLN235, ARG236  **π-π interaction:** HIE229  **Hydrophobic:** CYS237  **Polar:** THR221, HIS223, HIE229, GLN235  **Charged Positive:** LYS222, ARG236 | 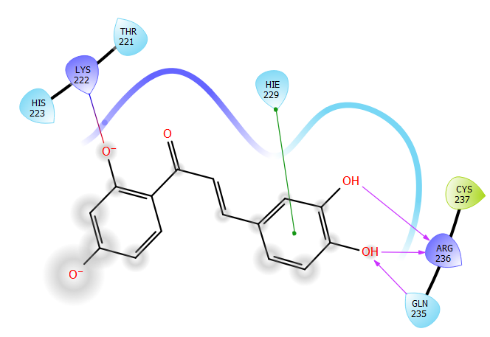 |
| ZINC000005842416 (Scutellarein) | -9.088 | -22.25 | **H-Bond:** HIS233, GLN235, ARG236  **Salt Bridge:** LYS222  **Hydrophobic:** CYS237, TYR238, CYS245  **Polar:** THR221, HIS223, HIE229, GLN235  **Charged Positive:** LYS222, ARG236 | 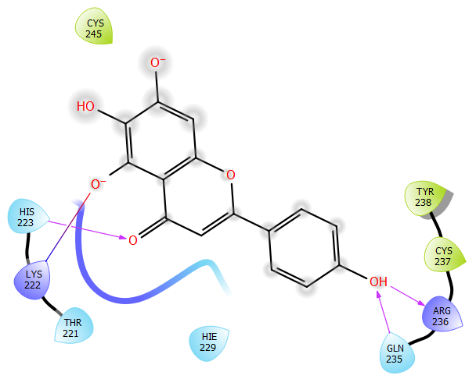 |

**Table 5:** Dock score, binding energy, and interacting residues for GSK-3β enzyme (PDB id: 1Q5K) of top five molecules.

| **Molecular docking in XP docking mode for GSK-3β (PDB id:1Q5K)** | | | | |
| --- | --- | --- | --- | --- |
| **Compound** | **XP Dock score (kcal/mol)** | **MMGBSA dG Bind**  **(kcal/mol)** | **Non-bonding interactions** | **2D Interaction diagram** |
| ZINC33832403  (Mangiferin) | -10.344 | -34.91 | **H-Bond:** ASP133, LYS183, ASN186, ASP200  **Hydrophobic:** ILE62, PHE67, VAL70, ALA83, VAL110, LEU132, TYR134, VAL135, LEU188, CYS199  **Polar:** SER66, GLN185, ASN186  **Charged Negative:** ASP133, ASP181, ASP200  **Charged Positive:** LYS85, LYS183 | 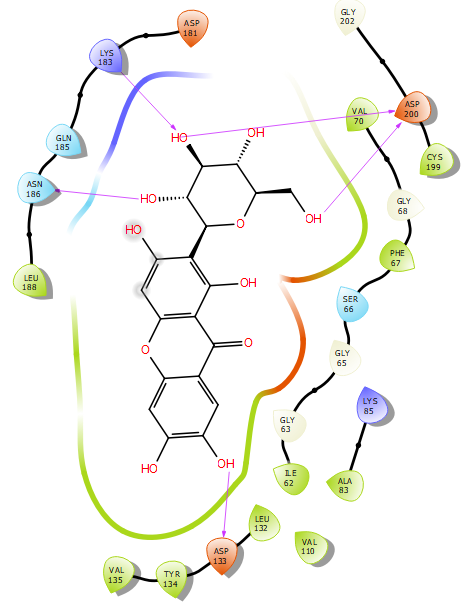 |
| ZINC3881558  (Morin) | -9.427 | -41.27 | **H-Bond:** VAL135  **Hydrophobic:** ILE62**,** VAL70, ALA83, VAL110, LEU132, TYR134, VAL135, PRO136, LEU188, CYS199,  **Polar:** THR138  **Charged Negative:** ASP133, GLU137, ASP200  **Charged Positive:** LYS85, ARG141 | 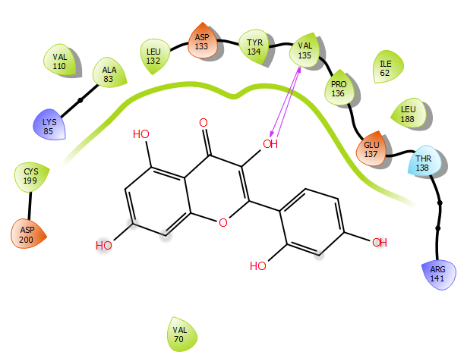 |
| ZINC100067274 (Curcumin) | -9.165 | -65.34 | **H-Bond:** LYS85, VAL135, ASN186, ASP200  **Hydrophobic:** ILE62, VAL70, ALA83, MET101, VAL110, LEU130, LEU132, TYR134, VAL135, LEU188, CYS199, PHE201  **Polar:** ASN64, THR138, GLN185, ASN186  **Charged Negative:** GLU97, ASP133, GLU137, ASP200  **Charged Positive:** LYS85 | 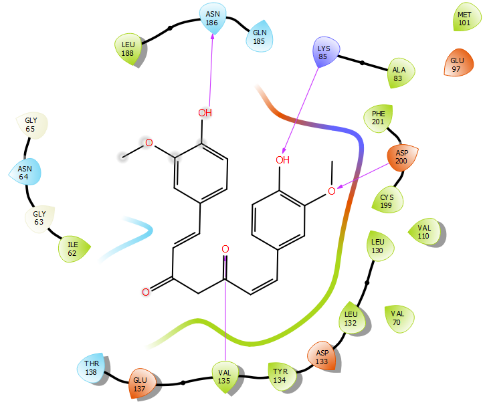 |
| ZINC3943903  (Baicalin) | -8.738 | -45.32 | **H-Bond:** ILE62, VAL135  **Hydrophobic:** ILE62, VAL70, ALA83, VAL110, LEU132, TYR134, VAL135, PRO136, TYR140, LEU188, CYS199, PHE201  **Polar:** THR138, GLN185  **Charged Negative:** GLU97, ASP133, GLU137, ASP200  **Charged Positive:** LYS85, ARG141, ARG144 | 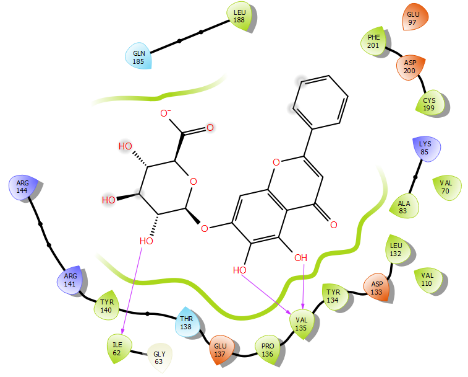 |
| ZINC1536 (Honokiol) | -8.556 | -54.74 | **H-Bond:** VAL135  **Hydrophobic:** ILE62, VAL70, ALA83, VAL110, LEU132, TYR134, VAL135, PRO136, LEU188, CYS199  **Polar:** GLN72, THR138  **Charged Negative:** GLU137, ASP133, ASP200  **Charged Positive:** LYS85, ARG141 | 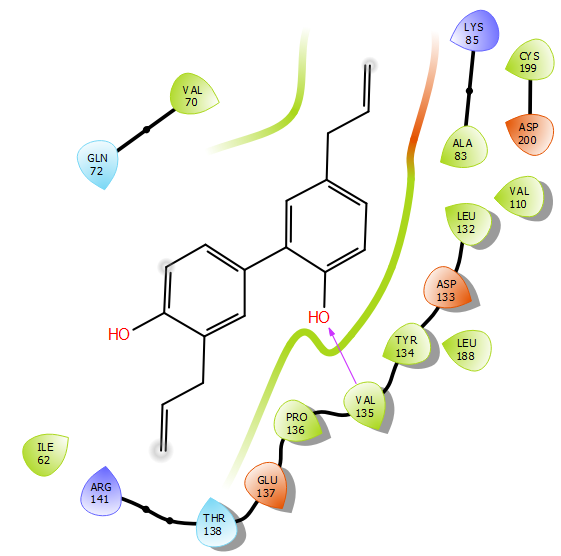 |

**Table 6:** Dock score, binding energy, and interacting residues for WIF1 protein (PDB id: 2YGO) of top five molecules.

| **Molecular docking in XP docking mode for WIF1 (PDB id:2YGO)** | | | | |
| --- | --- | --- | --- | --- |
| **Compound** | **XP Dock score (kcal/mol)** | **MMGBSA dG Bind (kcal/mol)** | **Non-bonding interactions** | **2D Interaction diagram** |
| ZINC33832403  (Mangiferin) | -13.546 | -53.75 | **H-Bond:** PRO78, THR167  **π-π interaction:** PHE89, PHE173  **Hydrophobic:** LEU38, ILE40, LEU48, ILE49, ILE57, MET63, PHE70, MET77, PRO78, ILE80, MET87, PHE89, PHE138, VAL136, PHE150, VAL152, VAL154, PRO168, PHE173,  **Polar:** THR167 | 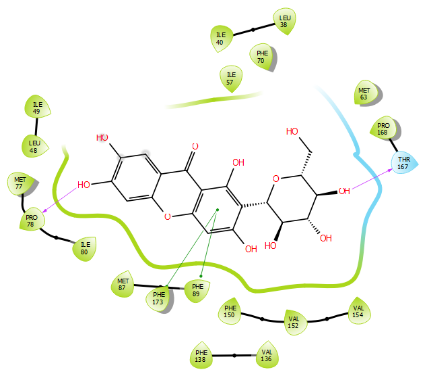 |
| ZINC103539689 | -12.667 | -60.85 | **π-π interaction:** PHE173  **Hydrophobic:** LEU38, ILE40, LEU48, ILE49, ILE55, ILE57, MET63, PHE66, PHE70, MET77, PRO78, ALA79, ILE80, MET87, PHE89, PHE103, VAL136, PHE138, PHE150, VAL152, VAL154, PRO168, PHE173, PHE174  **Polar:** THR167  **Charged Positive:** ARG76 | 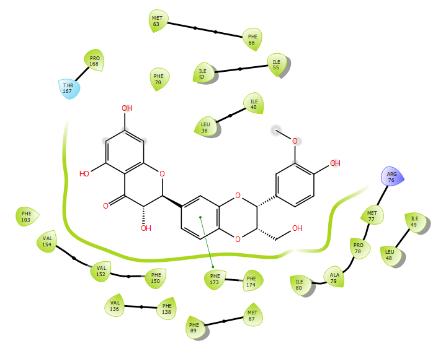 |
| ZINC100067274  (Curcumin) | -11.812 | -71.13 | **H-Bond:** PRO78  **π-π interaction:** PHE70  **Hydrophobic:** LEU38, ILE40, LEU48, ILE55, ILE57, MET63, PHE66, PHE70, MET77, PRO78, ALA79, ILE80, MET87, OHE89, VAL136, PHE138, PHE150, VAL152, VAL154, PHE173, PHE174,  **Polar:** THR67, THR167 | 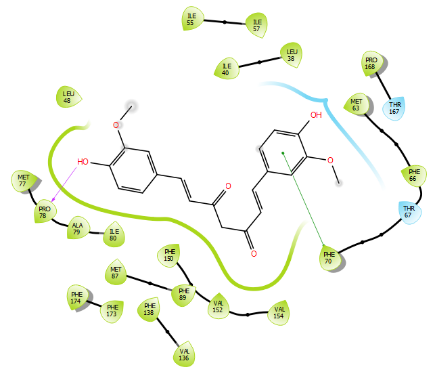 |
| ZINC3881558  (Morin) | -10.595 | -34.70 | **H-Bond:** PRO78, VAL152  **π-π interaction:** PHE173  **Hydrophobic:** ILE40, LEU48, ILE57, MET77, PRO78, ALA79, ILE80, MET87, PHE89, VAL136, PHE138, PHE173, PHE174, PHE150, VAL152, VAL154  **Polar:** THR167 | 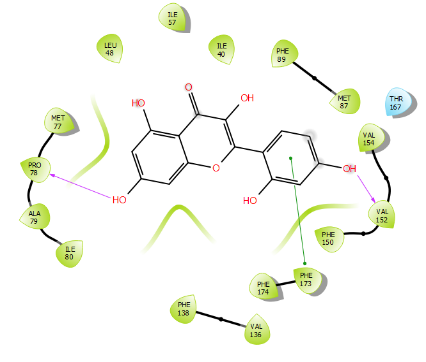 |
| ZINC5158604 (Amorphastilbol) | -10.308 | -60.35 | **Hydrophobic:** LEU36, TYR37, LEU38, ILE57, VAL58, MET63, PHE70, ILE80, PHE89, TRP91, TYR101, VAL127, VAL136, PHE138, PHE150, VAL152, VAL154, ILE155, VAL156, LEU165, PRO168, PHE173  **Polar:** THR67, GLN92, THR167 | 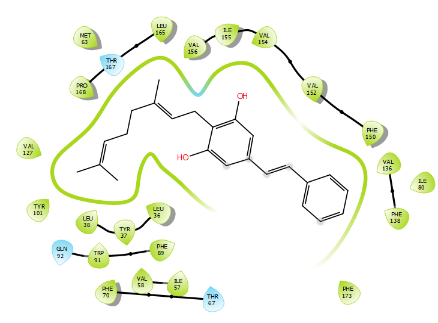 |
